# Supplementary material for: Are carbon emissions trading and green financial instruments synergistic? -Comprehensive quantitative research based on content analysis
Source: PLoS One. 2024 Mar 7;19(3):e0298601. doi: 10.1371/journal.pone.0298601 (PMC10919601; doi:10.1371/journal.pone.0298601)
Supplement: S1 Data — (ZIP) [file pone.0298601.s001.zip › Data/地级市Z_F工作报告.zip!/╜¡╦╒/│ú╓▌╩╨/└┤╫╘╣1⁄2╓┌║┼í╛┬φ┐╦╩2╛▌═°í┐_2010.docx]

各位代表：

　　现在，我代表常州市人民政府，向大会作工作报告，请予审议，并请市政协各位委员和其他列席人员提出意见。

　　一、2009年工作回顾

　　刚刚过去的2009年，是极不平凡的一年，历史罕见的国际金融危机给我市发展带来了前所未有的困难。市政府在中共常州市委的领导下，在市人大和市政协的监督支持下，深入学习实践科学发展观，坚定信心，积极应对，攻坚克难，负重奋进，着力做好保增长、保民生、保稳定各项工作，较好地完成了市十四届人大二次会议确定的目标任务。

　　（一）经济平稳较快增长。全年实现地区生产总值2518.9亿元，按可比价增长11.7%，户籍人均水平超过1万美元。地方一般预算收入突破200亿元，达到215.9亿元，增长16.6%。全社会固定资产投资完成1704.8亿元，增长17.7%。工业经济量质齐升。规模以上工业完成产值5978.7亿元，增长15%。五大产业完成产值3690.8亿元，增长24.1%。规模以上高新技术企业完成产值2887亿元，增长23.9%。服务业发展势头强劲。实现服务业增加值997.3亿元，按可比价增长14.1%。成交额超 50亿元的大市场达到10家，超百亿元的3家。创意产业实现销售63亿元。旅游总收入超过260亿元，接待国内外游客2270万人次。现代农业再创佳绩。建成标准粮田21.4万亩，新增高效种植业13.8万亩。水稻单产再创历史新高，连续7年位居全省第一。开放型经济难中求进。实际到账注册外资22.6亿美元、新增工商登记注册外资43.2亿美元，分别增长10.9%和16.1%。引进光宝科技、晶元光电等一批重大项目。全年完成进出口总额150.8亿美元，下降14.5%，其中出口108.7亿美元，下降18.0%。武进出口加工区获国务院批准并封关运作，海关、国检金坛办事机构建成运行。

　　（二）结构调整取得成效。产业结构进一步优化。服务业增加值增速快于地区生产总值和工业增加值增速，在三次产业中的比重达到39.6%，提高1.6个百分点。投资结构得到改善。高新技术产业、服务业投资增速均超过全社会固定资产投资增速，分别达到21.8%和19.1%。增长动力结构更趋合理。社会消费品零售总额达901.4亿元，增长18.9%。科技创新能力不断增强。研发投入占地区生产总值的比重达2%，企业技术研发经费投入93.7亿元，新标准认定省高新技术企业113家。时速250公里动车组高速转向架及应用项目、稻/麦秸秆人造板技术与产业化项目分获国家科技进步一、二等奖。常州科教城被批准为国家大学科技园。私营经济蓬勃发展。新增个体工商户3.3万户、私营企业8946户、注册资本262.3亿元，分别增长14.1％、26.2％和37.1％。资本结构得到提升。金融机构本外币存贷款余额分别达到3854.89亿元和2568.93亿元，增长34.2%和36.9%。成功发行常州投资集团、武进城投、常高新等3只企业债券，融资额达57亿元。天龙光电在创业板成功上市。成立江南银行、两家村镇银行和4家农村小额贷款公司。人才结构不断完善。引进海外人才467名，其中领军型创新创业人才98名，千名海外人才引进计划五年目标三年完成。

　　（三）民生继续得到改善。城乡居民收入稳步提高。城镇居民人均可支配收入23760元，增长10%。农民人均纯收入超过11000元，增长10.1%。就业形势基本稳定。城镇新增就业8.2万人，农村劳动力转移2.13万人，城镇登记失业率控制在4%以内。社会保障水平得到提高。养老、医疗、失业保险参保分别净增4.5万人、4.9万人和4.2万人，三大保险综合覆盖率达到97%以上。被征地农民基本纳入社会保险范围，新型农村养老保险覆盖率达到77.8%。新型农村合作医疗参保率达100%。市区低保、居家养老服务补贴、居家养老服务中心运行补贴和百岁老人营养补贴提标全部落实到位。卫生惠民成效明显。累计建成城区卫生服务中心20家、服务站63家，完成社区卫生服务信息化系统一期工程，甲型H1N1流感得到有效防控。住房保障力度加大。对符合条件的经济适用房申请家庭每户补贴8万元，经济适用房购房签约5000户，新增廉租住房家庭1953户，基本实现了廉租住房和经济适用房应保尽保。完成65万平方米老小区整治，建成停车泊位6800个。公交优先战略深入实施。开通快速公交二号线，初步形成快速公交骨架网络，空调公交车比例超过65%，公交出行比例达25.5%。和谐安民工程取得成效。完成56个老住宅小区安防设施改造提升，建成156个技防村（社区）。社会治安综合治理绩效名列全省第一，被评为“全国社会治安综合治理优秀市”。

　　（四）城乡面貌又有变化。现代化综合交通网络进一步完善。常州机场新航站楼主体工程开工建设。西绕城高速、泰州长江大桥南接线工程超额完成年度目标。京沪高铁、沪宁城际、宁杭铁路客运专线及配套场站建设顺利推进。全面建成长虹路、通江大道、录安洲港区疏港公路及239省道改造等道路工程。录安洲夹江港区一期工程及京杭运河东、西港区建成投运，新沟河延伸拓浚工程列入国家太湖治理重点项目。城市建设加快推进。北部新城全面启动，南部新城初具规模，完成东部地区发展规划研究。清潭路、吊桥路、月季路改造工程完工。开工建设青洋路高架。中吴大道实施城市化改造。建成江边污水厂二期、戚区污水厂二期等重点市政工程。电网建设投入超过20亿元，供电能力和安全性大幅提高。城市长效管理水平逐步提升。建成数字化城管三级平台和视频监控系统，数字化城管覆盖面积扩大到220平方公里。完成城市主要道路环境提升任务。环境质量不断改善。节能减排指标全面完成，国家生态市建设考核指标达标率上升到85.2%，金坛、武进通过全国生态市（区）验收。11个太湖考核断面达标率82%，滆湖清淤一期工程全面完成，市区又有6条支河达到水清标准。空气质量优良天数达到334天。实施城乡绿化“八大工程”，新增绿地2584.4公顷。东坡公园、圩墩遗址公园、恐龙谷温泉公园建成开放，“三河三园”景观河带基本建成。新农村建设扎实推进。新改建农村公路297公里，疏浚县乡河道171条，整治村镇河塘4798个。966个村达到“三清一绿”，450个村达到“五化三有”。

　　（五）各项事业协调发展。教育发展水平进一步提升。所有辖市区均达到省教育现代化标准。全市省级优质中小学、幼儿园达到347所，占比率和就读率分别达到60.6%、72.3%。公办高中全部成为省三星级以上学校。省级优质职业学校占比率和就读率分别达到75%、90%。文化事业繁荣兴旺。常州大剧院竣工投运，现代传媒中心开工建设。各类博物馆、纪念馆和图书馆全面免费开放，农家书屋实现全覆盖，送文化下乡1.3万场次。电影《邓稼先》、广播剧《军训日记》获全国“五个一”工程奖，动画电影《麋鹿王》获全国华表奖。初步完成青果巷历史文化街区保护规划。卫生事业持续发展。市第三人民医院门急诊大楼、市疾控中心综合楼竣工投用。市第七人民医院门急诊病房综合大楼开工建设。完成数字卫生信息系统一期工程。体育事业蓬勃发展。被评为全国体育先进单位。第17届省运会筹备工作有序开展。建成圩墩公园羽毛球基地、西林公园全民健身基地和飞龙体育主题公园等户外广场，体育中心综合服务大楼及清潭体育馆改造项目顺利完工。精神文明和民主法治建设得到加强，社会保持和谐稳定。援助四川灾后重建工作可望实现“三年任务两年完成”。与此同时，安全生产、双拥共建、人武人防、外事侨务、对台事务、审计统计、海关国检、药监质监、粮食供销、边防海事、档案地方志、防灾减灾、民族宗教、老龄、妇女儿童、人口和计划生育、残疾人事业、关心下一代等工作都取得了新成绩。

　　（六）发展环境不断优化。深入开展“服务企业关爱有加、支持企业克难求进”活动，先后出台促进五大产业、外贸出口、房地产业发展等多项政策措施，充分发挥政策在保增长中的驱动效应。全市落实各类财政优惠政策155亿元，企业固定资产购进税款抵扣达18.96亿元，为出口企业办理各类出口退免税89.14亿元。开展银企对接洽谈活动，达成634亿元贷款合同和意向协议。落实收费减负项目71项，在全省率先放开环评、建设审图和房屋测绘市场。完成“三合一”网络平台二期建设，全市45个部门的行政处罚、行政强制、行政检查实现网上运行。行政审批集中度达到78%，重点项目审批提速45%。建立土地执法监察长效管理机制，保障依法高效用地。大力推进依法行政，自觉接受市人大、政协和各民主党派的监督，609件建议和提案全部办结。荣获中国社科院2009年中国城市竞争力评比政府创新能力第一名。

　　各位代表，我们之所以能够战胜各种困难，有效应对国际金融危机的挑战，继续保持经济社会健康稳定发展的良好势头，是全市人民在中共常州市委的领导下，坚决贯彻中央和省一系列宏观经济政策，克难求进、团结拼搏的结果，也是同方方面面的关心和支持分不开的。在此，我代表市人民政府，向全市人民，向市人大代表、政协委员、各民主党派、工商联、各人民团体、离退休老同志、无党派及各界人士，向驻常部队指战员、武警官兵和全市公安干警，向部、省驻常单位和在常的中外投资者、科技工作者、全体务工人员，向所有关心、支持常州建设和发展的海内外朋友们，表示衷心的感谢和崇高的敬意!

　　在肯定成绩的同时，我们也清醒地认识到工作中存在的问题。由于受金融危机的严重影响，年初确定的外贸目标没有完成；经济发展中结构性、素质性问题依然存在；产业招商还缺乏有效举措，工业投入的质量和水平有待提高；广大人民群众的收入增长还不快，中低收入群体的生活水平有待提高；行政机关的效能不尽如人意，政府职能转变还要进一步加快。这些问题都要在今后工作中切实加以解决。

　　二、2010年目标任务

　　2010年，是实施“十一五”规划的最后一年，也是充满挑战、大有作为的一年。全年政府工作的指导思想是：以科学发展观为指导，高扬“创新、发展、提高”主旋律，突出“招商、引智、投入”重点，坚持在保增长中快转型，在快转型中保增长；坚持以增量投入促进存量调整，在调整中实现产业升级；坚持外向带动战略，全面提升对外开放水平；坚持全面协调可持续发展，建设幸福和谐常州；坚持以民为本民生优先，让人民群众共享科学发展的成果。

　　全市主要预期目标是：地区生产总值增长12%；地方一般预算收入增长12%；全社会固定资产投资增长18%，其中工业投资增长20%；社会消费品零售总额增长17%；外贸进出口总额增长10％以上；注册外资实际到账增长10%；全社会研究与开发经费支出占地区生产总值的比重达到2.2%；城镇居民人均可支配收入增长10%，农民人均纯收入增长10%；城镇登记失业率控制在4%左右；万元地区生产总值综合能耗下降3.5%，化学需氧量和二氧化硫排放量均削减1%；环境质量综合指数达到85分以上。

　　为实现上述目标，今年将着力抓好六项重点工作：

　　（一）调结构

　　围绕经济转型升级，加快结构调整，加快发展低碳经济和创新型经济，尽快壮大优势产业，不断增强我市的综合竞争力。

　　1．加快发展新兴产业。（1）深入推进五大产业振兴计划。实施输变电设备、工程机械及车辆、新能源等九个专项产业发展规划，进一步提升产业规模和发展水平。五大产业规模以上企业产值增长20%，占全市规模工业的比重超过60%。（2）继续加大有效投入。深入开展“有效投入提升年”活动，推进160项市重点项目，实施100项工业重点项目，培育200个工业新增长点，确保全市工业投入超千亿。（3）推动企业做强做大。引导企业通过资产重组、资本运作、品牌提升、与央企合作等方式，拉长产业链，加快形成产业联盟、企业集团。把输变电、轨道交通产业打造成国内行业第一品牌，五大产业形成营业收入超100亿元的企业3家、50－100亿元的6家。

　　2．突破发展现代服务业。（1）推进大市场和物流园区建设。重点抓好东南陶瓷城改扩建等十大重点市场建设，年成交额超100亿元以上市场达4个，50亿元以上的达到12个。建成中国物流常州综合物流中心一期、亚邦食品医药物流中心等4个物流园区，开工建设苏浙皖边界物流中心改扩建项目。（2）大力发展生产性服务业。围绕软件、创意、产品交易等产业，着力打造一批生产性服务业集聚区，重点建设津通现代服务业交易中心。（3）提升旅游业竞争力。建设环球恐龙城迪诺创意园、环球数字狂欢谷等十大文化旅游项目，打造历史文化之旅。确保全年接待国内外游客2500万人次，旅游总收入突破300亿元。（4）创新金融服务。深入推进金融生态环境建设，所有辖市区均创建成省金融生态优良县（区）。进一步扩大信贷规模，确保信贷投放增长15％以上，中小企业贷款增长18％以上。组建3家农村小贷公司，引进1家外地金融机构，确保紫金保险常州公司开业运营。推进企业上市，力争2－3家企业在境内外上市。（5）促进房地产市场稳定健康发展。保持房地产消费政策的连续性，进一步规范和完善土地市场，稳定市场心理预期和房价。（6）建设新型商业街区。重点推进新北区通江中路、武进花园街、钟楼宝龙城市广场等五大新型商业街区建设。

　　3．提升发展高效农业。（1）深入实施现代农业“双百万亩”和“万顷良田”建设工程。新建10万亩高标准农田，新增10万亩高效种植业、3万亩高效渔业、3只亿元产出农产品。（2）加快现代农业示范园区建设。重点建设10个现代农业示范园区、10个高效设施农业示范基地、10个农业生态示范点和10个“一村一品”专业村，建设一批休闲观光农业发展集聚区。（3）推进农村改革富民。新发展农民专业合作社100家，组建专业合作联社5家。新建土地股份合作社20家，新增土地流转面积5万亩。新增农业适度规模经营面积20万亩。抓好帮扶茅山老区长效机制的落实，年收入20万元以下的经济薄弱村减少100个。全面完成集体林权制度改革。

　　4．大力发展开放型经济。（1）提升利用外资水平。加大招商引资力度，优化利用外资结构，突破外资大项目。新增总投资3亿美元以上项目2个、总投资超亿美元或工商登记注册外资超3000万美元项目18个、世界500强投资项目5个，制造业实际到账外资占全市65%，生产性服务业利用外资占服务业30%以上。（2）强攻突破外贸出口。全力拓展国际市场，加快培育规模企业，着力调整出口结构。新增出口超5000万美元企业5家、超亿美元企业2家，高新技术和机电产品出口占比分别提升3个百分点。（3）稳步推进企业“走出去”。新核准境外投资企业和中方协议投资均增长10%以上，新签外经合同和外经营业额均增长10％以上。（4）大力发展服务外包。以创意产业为重点，吸引境内外大型外包企业来常设立分支机构，培育一批领军型服务外包企业，争创全国服务外包示范城市。

　　（二）抓创新

　　以科技创新为核心，加快建设创新型城市，增强经济发展的内生动力，这是我市率先基本实现现代化的一项战略任务。

　　1．推进以企业为主体的创新体系建设。（1）大力发展高新技术产业。以企业牵引、产品带动，加快高新技术产业集聚扩张。年内重点培育省级高新技术产品150只，通过新标准认定省高新技术企业80家以上，全市规模以上高新技术产业产值增长18%以上。（2）突出企业自主创新。完善增强企业自主创新能力的政策体系和激励机制，激发企业创新热情，重点支持100家创新型试点企业，全市企业研发投入达到110亿元以上。（3）推进产学研合作。充分发挥在常高校的积极作用，大力引进大学大院大所来常设立研发机构，鼓励支持企业建设产学研合作基地，促进科技成果转移转化。全年引进研发机构5家，建立50家企业产学研合作基地和研发中心，实施产学研合作项目200项。（4）加强知识产权工作。专利申请量和授权量分别达10000件和3200件，其中发明专利申请1600件。

　　2．构建三级创新研发平台。（1）加快科教城建设。加快科教城二期及三期国际创新基地建设，科教城研发及孵化用房实现翻番，累计引进研发机构及高科技企业420家、研发人才8000人。（2）加快孵化器建设。全市孵化器面积累计达250万平方米，在孵企业3000家，1-2家孵化器进入国家级加速器试点。（3）加快企业研发机构和公共科技服务平台建设。新增企业创新平台60家，其中省级以上“两站三中心” 15家。培育市级以上科技公共服务平台10家，新增3-5家重大公共技术服务平台。

　　3．大力度引进高层次人才。（1）实施新一轮千名海外人才集聚工程。全年引进领军型创新创业人才100名、高层次研发人才100名、海外人才500名。（2）为高层次人才营造良好发展环境。加大政策优惠力度，加快领军型创新创业人才融资平台和特色孵化器等载体建设，为海内外人才创新创业提供良好的条件。

　　4．全面推进创新型科技园区建设。（1）科学制订园区发展规划。充分利用国家高新区、武进高新区和常州科教城的创新资源，以五大产业为重点，努力建设创意、光伏、风电设备、生物医药、新能源汽车、机器人及智能装备、半导体照明和功能材料等八大创新产业集群，使创新型科技园区成为常州可持续发展的创新动力源和经济增长极。（2）整合资源形成合力。整合全市科技、教育、人才和产业资源，调动“两区一城”的创新积极性，力争通过5年努力，创建成国家创新型科技园区。（3）促进全市园区加快发展。全年新增开发区基础设施投入100亿元，开发区实际到账外资、地区生产总值、一般预算收入占全市比重分别超过75%、45%和50%，新增外资制造业大项目占全市80%以上，单位面积投资强度和产出水平均达300万元/亩以上。

　　（三）重民生

　　坚持把民生工作摆在更加突出的位置，全心全意为人民谋利益，努力使常州人民的生活一年更比一年好。

　　1．以创业带动就业。（1）推动全民创业。完善创业扶持体系，激发全民创业热情，扩大创业带动效应。全年新增个体工商户1.8万户、私营企业7200家、个私注册资本120亿元。用三年时间，建立创业孵化基地50个，扶持创业1万人，带动就业5万人。（2）努力扩大就业。通过完善政策、资金扶持、市场对接、培训提升、优化服务等措施，稳定扩大就业。挖掘和提供公益岗位，帮助弱势群体解决就业困难。

　　2．提高社会保障水平。（1）完善城乡居民社会养老保障体系。建立市区城镇居民社会养老保险制度，完善新型农村养老保险，做好城保、新农保的制度衔接工作。（2）进一步提高社会保障水平。城镇养老、医疗、失业保险分别净增参保3万、3万、2万人，三大保险综合覆盖率保持在97%以上。基本实现农村社会养老保险应保尽保。继续增加企业退休人员养老金。（3）实现“小康常州，居者有其屋”的目标。适当降低住房保障申请条件，扩大住房保障覆盖面。廉租住房和经济适用住房保障做到应保尽保。

　　3．优化社会公共服务。（1）继续实施公交优先发展战略。推进城乡公交一体化，市区行政村公交通达率达100％。加快建设公交调度指挥中心和城铁、高铁公交枢纽站。新建候车亭300座。新购空调公交车300辆，市区空调车比例达70％以上。（2）大力发展公用事业。实施500千伏和220千伏输变电工程建设。新建燃气管网50.4公里。加强饮用水源保护和饮用水监测，确保居民饮用水质量。开工建设江边污水处理厂三期工程，新建30公里污水管网。（3）推进菜市场改造提升。改造提升4家菜市场。加强农副产品安全检测，完善菜市场长效管理机制。(4)全面完成公厕改造提升任务。新建公厕23座、改造46座。

　　4．继续办好十件实事。（1）扩大就业。全年新增就业岗位8万个,新增就业6万人,援助困难群体就业3000人,动态消除城镇零就业家庭和农村零转移家庭。（2）新农保扩面。年内全市新型农村社会养老保险覆盖面达到90%以上，武进区、新北区农村老年居民补贴标准每人每月60元。（3）住房保障。续建廉租住房和公共租赁住房20万平方米，新开工建设12万平方米，实物配租500户。蓝天新苑、飞龙新苑经济适用房工程一期34万平方米主体竣工。住房公积金扩面5.5万人以上，发放低息贷款20亿元。（4）对老住宅小区进行全面整治和提升。非成建制老住宅小区：年内完成125万平方米，达到“基本设施完善、住宅环境整洁、实现物业管理”。成建制老住宅小区：年内完成市区65万平方米，达到“立面出新、楼道亮化、环境美化、安防设施完善、物业管理和谐”。在30个小区增设停车泊位5000个。（5）提升医疗保障水平。城镇职工和居民医保最高支付限额分别提高到职工年平均工资和居民年人均可支配收入6倍以上，制度规定范围内医药费用报销比例分别达到80％和60％。各级财政对居民医保的补贴标准每人每年不低于120元。（6）爱老助老。民办投资建设的养老机构新增床位补贴标准提高到3000-4000元，租赁创办的新增床位每张补贴1000元。对市区90周岁以上老人每月补贴200元，其中全护理人员再提高100元。建设市爱心护理院，设置200张床位。建设数字化为老服务平台，逐步打造“十五分钟养老服务圈”。（7）残疾人服务“三进五有一免”工程。推进残疾人“康复服务、安养服务、无障碍设施进家庭”，实现残疾人“有康复中心、就业教育培训中心、庇护安养机构、辅助器具服务中心、文化体育活动中心”，市区持证残疾人免费乘坐公交车。为3900名有康复需求的残疾人提供康复服务，为628名低保重度残疾人实施居家安养服务，为300 户有需求的困难家庭装配无障碍设施。建成2万平方米市康复中心、1600平方米教育就业技能培训中心、5000平方米全日制托养服务中心、800平方米辅助器具服务中心、1800平方米文化体育活动中心。（8）和谐安民提升工程。对1998年后建的100个老住宅小区和125万平方米散居楼进行整治，全面安装电子防盗门，新增防爬套，新建围墙，增设门岗，新增社会面和老住宅小区视频监控探头850个，建成46个技防村（社区），完成技防入户2万家。（9）提升农村小康水平。完成农村实用技术、实用人才等培训10万人。新农合参保率达100%,人均筹资达240元，住院补偿率达到45％。每镇每季送一场戏，每村每月送一场电影，每镇每年送价值1万元的书籍，新增农村有线电视用户2.5万户。建设为农服务社200家，为农服务社行政村覆盖率达到90％。（10）建设宜居环境。绿地：年内建成或基本建成城铁北广场绿地、雕庄公园、丁塘港生态湿地公园等9个公园绿地，启动圩墩公园三期、桃园绿地二期等7个公园绿地建设。碧水：继续推进市区60条河道清水工程，巩固提升52条河道水质，综合整治8条河道水环境，完成10条河道生态修复。蓝天：重点对龙虎塘、新闸、牛塘、滨江等4个区域的工业企业进行恶臭气体整治，20家重污染企业实施限停产或驻厂监管。“禁燃区”建设扩大至66平方公里，积极推进秸秆综合利用，全面实施高污染车辆限行管理，整治扬尘污染。全年空气质量优良天数达到330天以上。

　　（四）优环境

　　按照“民本、现代、文化、生态”理念，加快城市化和国家生态市建设步伐，全面优化人居环境，全方位展示常州对外新形象。

　　1．加快基础设施建设。（1）推进铁路站场建设。建成常州市客运中心及综合配套系统、沪宁城际铁路戚墅堰站，完成京沪高速铁路常州站、宁杭铁路客运专线溧阳站、瓦屋山站主体工程。（2）加快建设以高速公路、干线公路为主的公路网络。续建常州西绕城高速公路、泰州大桥接线常州段、104国道溧阳西段、241省道金坛北段。开工建设苏锡常南部通道常州段、常溧高速公路。（3）实施机场改扩建及配套道路工程。完成机场改扩建工程，常州机场高架连接线、新机场路城市化改造建成通车。（4）加快港口航道建设。建成录安洲港区1号泊位。加快苏南运河西段、锡溧漕河、芜申线、丹金溧漕河等三级航道网整治。（5）推进水利工程建设。开工建设城市防洪大运河东枢纽、南运河枢纽、采菱港枢纽等节点工程，完成沙河水库、大溪水库、前宋水库的除险加固，积极推进新孟河、新沟河延伸拓浚工程。

　　2.统筹推进城乡建设。（1）完善中心城区路网。完成青洋路高架、中吴大道城市化改造工程，实施劳动东路东段、白云南路等道路建设工程。戚月路及地道、永宁路及地道和新堂北路、竹林西路、飞龙东路建成通车。（2）加快建设南北新城区。南部新城新建续建延政西路、聚湖西路等骨干道路，实施兰陵南路全面改造，加快西太湖生态休闲区建设。北部新城完善重点地区规划，建设创意产业基地、高铁场站周边等重点片区路网。（3）实施东大门三期建设工程。建成常焦线、泡桐路武进段，开工建设工业大道、大明路北段，完成东方东路城市化改造、革新河清水工程建设。（4）增强城镇承载能力。按照统筹规划、合理布局、完善功能、辐射带动的原则，加快金坛、溧阳城市化建设步伐，集中力量抓好一批在区位、产业等方面有明显优势的重点中心镇建设，增强其吸纳人口和就业的能力。（5）实施农村环境提升工程。在全面完成“三清一绿”、“五化三有”的基础上，以辖市区为单位，建立村庄环境长效管理机制。新建农村公路60公里、桥梁100座。完成50个行政村、28条县乡河道、728个河塘整治任务。

　　3．改善生态居住环境。（1）重点实施五大特色绿化工程。一是公园绿地建设工程，办好2010年第四届中国月季花展暨世界月季联合会区域性大会。二是道口绿化工程，对沪宁高速常州、横山桥、薛家道口、沿江高速常州南道口进行绿化提升，对沿江高速滆湖道口进行绿化。三是垂直绿化工程，对铁路地道、高架路桥柱、部分河道进行垂直绿化。四是道路绿化工程，全面提升现有道路绿化水平，城区的主要道路不但要全年常绿，还要实现四季有花。五是围墙绿化工程，对146公里围墙实施绿化。全年新增绿化面积553.47公顷。（2）巩固提升水环境质量。完成太湖水污染防治年度目标，3条入太湖河道主要水质指标年均值达到五类水质标准。（3）加大节能减排力度。强化重点用能企业管理，发挥公共机构节能表率作用。做好江边污水处理厂等大型污水处理厂减排后续项目，开展城市污水处理厂中水回用。加快金坛、溧阳、武进乡镇污水处理厂管网建设。完成工业固体废弃物安全填埋场一期工程主体建设。

　　4．全面提升长效管理。（1）提升数字化城管水平。进一步细化主城区内的基础数据普查，对街巷里弄等未普查区域进行补测，实现数字化管理全覆盖。（2）加强市容环境整治和管理。对沪宁城际铁路、青洋路快速高架两侧进行整治。对中吴大道、飞龙东路等22条城市主要道路进行环境提升。（3）实施“一路两区”亮化工程。全面完成通江大道-怀德路-延陵路-和平北路一线两侧和奥体中心片区、南部新城片区的亮化提升工作。（4）动员全市人民参与城管。组织我爱我家城市管理百人“找差团”活动，邀请人大代表、政协委员和市民网民代表与城管部门一起，查找城市管理的薄弱环节，制定落实整改方案，全面提升城管水平。

　　（五）促和谐

　　加快发展社会事业是促进科学发展的重要举措，要加大投入力度，优化资源配置，着力提高社会管理和公共服务水平。

　　1．提升教育整体水平。（1）推进教育重大项目建设。实施常州技师学院新校区、刘国钧高职校、市五中、北郊中学初中部等21个千万元以上重点项目，完成35万平方米校舍抗震改造建设任务。（2）促进教育均衡发展。新增省优质幼儿园5所、优质小学18所、优质初中7所。外来务工人员子女接受义务教育普及率100％，公办学校吸纳比例85％以上。（3）全面实施素质教育。切实推进减负增效，新增德育、科技、体育、艺术等特色学校100所。

　　2．提高卫生服务质量。（1）加快医疗机构基础建设。市中医院门诊病房综合楼、阳湖医院竣工投运，完成三院院区和卫生监督所改造。实施儿童医院病房楼装饰、七院综合楼建设，完善数字卫生信息化系统一期工程。（2）减轻群众就医负担。积极推进医药卫生体制改革，切实降低人民群众就医费用。大力发展社区卫生，城市社区卫生服务机构建设全面达标，医院挂钩帮扶社区卫生服务机构覆盖率达100%，对社区卫生机构继续实行“六免二减”政策和基本药品零差率销售。（3）实施重大公共卫生服务项目。加强甲型H1N1流感等疾病的防控，加大基本公共卫生投入，全市人均基本公共卫生服务经费标准提高到农村15元、城市20元。

　　3．发展繁荣文化事业。（1）加快文化重点项目建设。加快建设常州未成年人社会实践基地，实施青果巷历史文化街区保护工程，全面推进历史文化名城建设。（2）加强文化基础设施建设。全面提升数字电视服务质量，提升乡镇文化站和行政村农家书屋水平，农村行政村文化室全面达标。（3）开展群众性文化活动。常州大剧院演出超过100场，广场、书场、小剧场等演出1000场次以上。

　　4．办好十七届省运会。（1）当好东道主。认真做好筹备和组织工作，以“全新的城市形象、现代的比赛场馆、一流的组织水平、精彩的开幕闭幕、辉煌的竞技体育、热情的文明市民”，全面展示常州的对外形象，把十七届省运会办成江苏历史上最精彩、圆满、成功、难忘的运动会。（2）以迎接十七届省运会为契机，全面实施《全民健身条例》。完善和提升新建小区、老小区的体育设施。扩大奥体中心、中天体育馆开放项目。举办好中国羽毛球大师赛、世界跳水系列赛等重大国际赛事。

　　5．协调发展各项事业。加大全国文明城市创建力度，不断提升全民素质。认真编制“十二五”规划纲要。健全应对突发事件的工作机制，全力做好安全生产长效管理和重大危险源监控，实施食品药品安全放心工程。认真做好信访工作，妥善化解社会矛盾。做好第六次全国人口普查，深入推进普法工作。加强国防教育、国防动员和人民防空工作，扎实推进双拥共建活动。推行优生优育早教工程，实现人口计生优质服务体系城乡全覆盖。支持工会、共青团、妇联等人民团体工作，进一步加强外事、侨务、台湾事务、地方志、民族宗教、防灾减灾、关心下一代、妇女儿童等工作。

　　（六）强自身

　　新形势、新任务对政府工作提出了新的更高的要求，要以转变政府职能为核心，着力提高公务员的整体素质和服务水平。

　　1．推进改革。（1）推进机构改革。完成市县政府和乡镇机构改革，理顺职责关系，将政府职能切实转移到为市场主体服务和创造良好发展环境上来。（2）深化行政审批制度改革。逐步做到市级机关行政审批职能向一个处室集中，审批处室向行政服务中心集中。行政审批事项集中度提高到82％以上，“一事一评”满意度稳定在99%以上。（3）完善公共财政制度。统筹安排预算内外资金，建立国库单一账户体系，规范预算单位账户管理。推进政府非税收入改革，健全政府投资管理体制，提高财政资金使用效益。

　　2．崇尚创新。（1）树立改革创新意识。一切从有利于发展出发，一切从方便企业和市民出发，创造性地开展工作，让常州的发展永远充满生机和活力。（2）创新政府管理和服务方式。加快电子政务建设进程，完善“三合一”网络平台，实现省、市、县三级全面联网交换数据。各部门要围绕发展中的重点、难点、热点问题，勇于突破，敢于创新。（3）全面推进政务公开。完善重大事项集体决策、专家咨询、社会公示与听证等制度，有效扩大公众对政府决策的参与度。健全政府信息发布和发言人制度，发挥好市长公开电话、市长与网民交流平台和部门服务热线作用，及时解决人民群众的合理诉求。

　　3．重在落实。（1）转变作风。全体公务员要深入实际，服务企业，服务群众，工作要雷厉风行，说干就干，一抓到底。（2）强化监督。自觉接受人大和政协的监督，自觉接受群众监督和舆论监督，支持监察、审计等部门依法履行监督职责。（3）依法问责。坚持依法、规范、高效行政，严格、公正、文明执法。对不作为、乱作为行为，实行严格的问责制度。

　　4．廉洁从政。（1）坚持廉洁自律。全体公务员要严格要求自己，自觉遵守廉洁自律的各项规定，始终保持为民务实清廉的良好形象。（2）严惩违法犯罪。加大违法违纪案件查办力度，坚决打击贪污贿赂、腐化堕落等违法犯罪行为。（3）强化制度建设。深化土地出让、工程招标、产权交易、政府采购等重点领域的廉政体系建设，推进公共资源市场化配置。（4）节约行政开支。严格控制公用经费和一般性支出，坚决制止铺张浪费，确保把有限的资金用于加快发展和改善民生。

　　各位代表，新的历史征程呼唤着我们，新的宏伟目标激励着我们。让我们在中共常州市委的领导下，团结一致，坚定信心，锐意进取，开拓创新，为率先基本实现现代化、再创常州新辉煌而努力奋斗！
